# Genome analysis and *in vivo* virulence of porcine extraintestinal pathogenic *Escherichia coli* strain PCN033

**DOI:** 10.1186/s12864-015-1890-9

**Published:** 2015-09-21

**Authors:** Canying Liu, Huajun Zheng, Minjun Yang, Zhuofei Xu, Xiangru Wang, Liuya Wei, Biao Tang, Feng Liu, Yanyan Zhang, Yi Ding, Xibiao Tang, Bin Wu, Timothy J. Johnson, Huanchun Chen, Chen Tan

**Affiliations:** State Key Laboratory of Agricultural Microbiology, College of Veterinary Medicine, Huazhong Agricultural University, Wuhan, China; Department of Veterinary Medicine, Foshan University, Foshan, Guangdong China; Shanghai-Most Key Laboratory of Health and Disease Genomics, Chinese National Human Genome Center at Shanghai, Shanghai, China; State Key Laboratory of Genetic Engineering, Department of Microbiology, School of Life Sciences, Fudan University, Shanghai, China; Department of Veterinary and Biomedical Sciences, University of Minnesota, Saint Paul, Minnesota USA

**Keywords:** Porcine extraintestinal pathogenic *Escherichia coli*, Comparative genomic analysis, Pathogenicity analyses, Virulence factors

## Abstract

**Background:**

Strains of extraintestinal pathogenic *Escherichia coli* (ExPEC) can invade and colonize extraintestinal sites and cause a wide range of infections. Genomic analysis of ExPEC has mainly focused on isolates of human and avian origins, with porcine ExPEC isolates yet to be sequenced. To better understand the genomic attributes underlying the pathogenicity of porcine ExPEC, we isolated two *E. coli* strains PCN033 and PCN061 from pigs, assessed their *in vivo* virulence, and completed and compared their genomes.

**Results:**

Animal experiments demonstrated that strain PCN033, but not PCN061, was pathogenic in a pig model. The chromosome of PCN033 was 384 kb larger than that of PCN061. Among the PCN033-specific sequences, genes encoding adhesins, unique lipopolysaccharide, unique capsular polysaccharide, iron acquisition and transport systems, and metabolism were identified. Additionally, a large plasmid PCN033p3 harboring many typical ExPEC virulence factors was identified in PCN033. Based on the genetic variation between PCN033 and PCN061, corresponding phenotypic differences in flagellum-dependent swarming motility and metabolism were verified. Furthermore, the comparative genomic analyses showed that the PCN033 genome shared many similarities with genomic sequences of human ExPEC strains. Additionally, comparison of PCN033 genome with other nine characteristic *E. coli* genomes revealed 425 PCN033-special coding sequences. Genes of this subset included those encoding type I restriction-modification (R-M) system, type VI secretion system (T6SS) and membrane-associated proteins.

**Conclusions:**

The genetic and phenotypic differences between PCN033 and PCN061 could partially explain their differences in virulence, and also provide insight towards the molecular mechanisms of porcine ExPEC infections. Additionally, the similarities between the genomes of PCN033 and human ExPEC strains suggest that some connections between porcine and human ExPEC strains exist. The first completed genomic sequence for porcine ExPEC and the genomic differences identified by comparative analyses provide a baseline understanding of porcine ExPEC genetics and lay the foundation for their further study.

**Electronic supplementary material:**

The online version of this article (doi:10.1186/s12864-015-1890-9) contains supplementary material, which is available to authorized users.

## Background

*Escherichia coli* is a well-known prokaryotic organism that can exist both as a harmless intestinal inhabitant and a deadly pathogen [1]. Pathogenic *E. coli* can be divided to intestinal pathogenic *E. coli* (IPEC) and ExPEC [2]. ExPEC strains harbor diverse genomes and a wide range of virulence factors (VFs) that collectively confer the ability to colonize and invade extraintestinal sites, causing infections of the urinary tract, meningitis, pneumonia, osteomyelitis, and surgical site infections [3]. ExPEC strains mainly include uropathogenic *E. coli* (UPEC), newborn meningitis-causing *E. coli* (NMEC), and avian pathogenic *E. coli* (APEC) [1]. Infections caused by ExPEC strains occur worldwide and incur great economic cost [4].

The major VFs present in ExPEC strains are distinct from those found in IPEC strains. Characteristic ExPEC VFs include various adhesins (P and type I fimbriae), structural components of the bacterial outer membrane (capsule, lipopolysaccharide), iron acquisition and utilization systems (aerobactin and salmochelin siderophores), toxins (hemolysin, cytotoxic necrosis factor) and secretion systems [4, 5]. These VFs are frequently encoded on mobile genetic elements in unique organization [1]. For example, possession of ColV plasmids are a defining trait of APEC and these plasmids are key players in APEC pathogenesis [6].

Some virulence-associated genes are shared between ExPEC independent of host species, suggesting that ExPEC may be zoonotically acquired [7]. Previous studies have shown that some APEC strains shared similarities with human ExPEC strains, and a foodborne link between the two may exist [8]. To date, twelve and two complete genomic sequences of human ExPEC and APEC strains are respectively available in GenBank (Additional file 1: Table S1); however, no porcine ExPEC have been sequenced yet. Recently, ExPEC infections have become epidemic in the Chinese pig industry, and these ExPEC could represent a public health threat [9]. Therefore, a better understanding of porcine-source ExPEC genomics is needed to improve our understanding of its mechanisms of disease. In this study, PCN033 was chosen for study as its serotype O11 and phylogenetic group D were characteristic of porcine ExPEC strains [9–11]. *E. coli* strain PCN061, which was also isolated from extraintestinal site of diseased pig, was used to perform comparative genomic analyses with PCN033. Pig experiments were then conducted to compare the pathogenicity of these strains in an extraintestinal disease model. Additionally, comparative genomic analysis was conducted among PCN033 and other nine characteristic *E. coli* strains to identify genomic differences that may explain the ability of PCN033 to cause disease in the pig host.

## Result and discussion

### Choice of strain

**Fig. 1:**
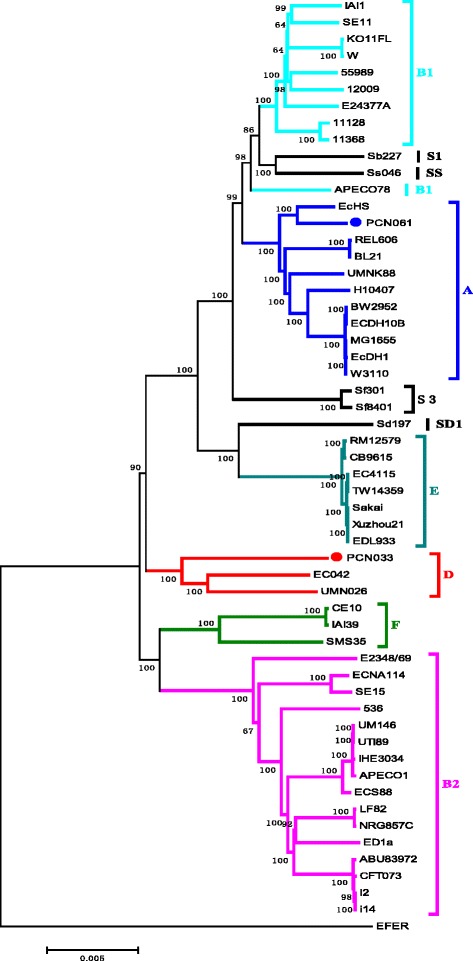
The phylogenetic analysis of *E. coli/ Shigella*. The phylogeny was constructed using the concatenated 325 genes conserved in all 56 fully sequenced *Escherichieae* strains. *Escherichia fergusonii* (ATCC 35469) was used as an out-group. The phylogroup (A, B1, B2, D, E, F, S1, S3, SS or SD1) of each strain is indicated on the right. The numbers near individual branches indicate the bootstrap percentage of 100 replications. Full names and accession numbers for selected bacterial genome sequences are listed in Additional file 1: Table S1. The 325 core genes of the 56 analyzed Escherichia strains were listed in Additional file 2: Table S2.

Our lab previously performed an epidemiological analysis of porcine ExPEC in China [9, 11]. Among the porcine *E. coli* isolates, PCN033 was selected [10] as it was defined as a porcine ExPEC strain follow the criterion by Johnson et al. [12] and Ding et al. [11]. PCN033 contained two of the ExPEC virulence markers, *kpsMTII* and *iutA*. Furthermore, it belonged to O11 serogroup which is one of the most prevalent among porcine ExPEC in China [9]. Additionally, PCN033 belongs to phylogenetic group D (Fig. 1). Porcine ExPEC are mostly fell in phylogenetic groups A, B1 and D [11]. More importantly, PCN033 presented as a highly virulent ExPEC strain in previous virulence assessment in the mouse model [9, 13]. In sum, PCN033 possessed characteristic traits of porcine ExPEC. Another porcine *E. coli* isolate PCN061 was chosen to perform comparative genomic analyses with PCN033 as it was also isolated from extraintestinal site of pig but contained none of the ExPEC virulence markers. PCN061 belongs to phylogenetic group A which is representaive of porcine *E. coli* strains. It was an O9 strain.

### Pathogenicity analysis

Four of the piglets in PCN033 group showed severe clinical signs, such as lying on the side, abdominal breathing, shaking, convulsion, lameness etc. and died successively within 5 days post-inoculation. All piglets in PCN061 group survived. Piglets in negative control group were all in good condition until euthanasia (Table 1). Meanwhile, our bacterial recovery test showed that the inoculated bacteria were re-isolated from blood samples of PCN033 group, however in PCN061 and negative control group, bacteria were not recovered. The test showed that PCN033 could cause pathological conditions and even death in pig model.

**Table 1:** The mortality of piglets after infection

Dosage	Inoculum	Animals with serious symptoms^a^	Days after infection until dead or euthanasia^b^	Animals surviving^c^
8 × 10^8^ CFU (1 ml)	PCN033	4/6	1, 2, 2, 5, 7, 7	2/6
8 × 10^8^ CFU (1 ml)	PCN061	0/6	7, 7, 7, 7, 7, 7	6/6
1 ml	PBS	0/3	7, 7, 7, 7, 7, 7	3/3

^a^Results are indicated as the number of piglets with serious symptoms out of the total piglets in each group

^b^Any piglets survived until the day 7 were euthanized

^c^Results are indicated as the surviving number of piglets out of the total piglets in each group

### General features of PCN033 and PCN061 genomes

**Fig. 2:**
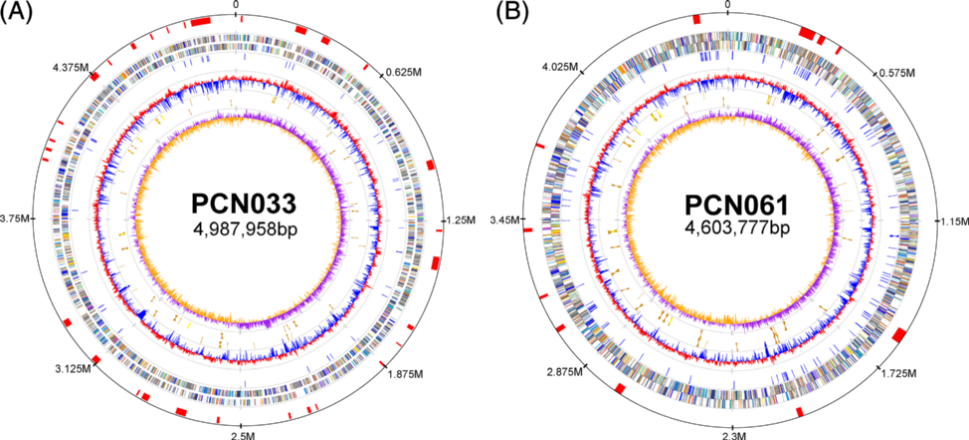
Circular maps of PCN033 and PCN061 chromosome. Circles are numbered from 1 (outer circle) to 8 (inner circle). Circle 1, predicted genomic island in red. Circles 2/3 shows predicted CDSs on the plus and minus strand color-coded by COG categories. All genes are colored according to biological functions: gold for translation, ribosomal structure and biogenesis; orange for RNA processing and modification; light orange for transcription; dark orange for DNA replication, recombination and repair; antique white for cell division and chromosome partitioning; pink for defense mechanisms; tomato for signal transduction mechanisms; peach for cell envelope biogenesis and outer membrane; deep pink for intracellular trafficking, secretion and vesicular transport; pale green for posttranslational modification, protein turnover and chaperones; royal blue for energy production and conversion; blue for carbohydrate transport and metabolism; dodger blue for amino acid transport and metabolism; light blue for coenzyme metabolism; cyan for lipid metabolism; medium purple for inorganic ion transport and metabolism; aquamarine for secondary metabolites biosynthesis, transport and catabolism; gray for function unknown. Circle 4, predicted insertion sequence elements in blue. Circle 5, mean centered GC content (red: above mean, blue: below mean). Circle 6/7 shows predicted RNAs on the plus and minus strand, orange for tRNA, yellow for rRNA. Circle 8, Gcskew plot (windowsize: 1000, windowoverlap: 500). **a** Circular map of PCN033 chromosome. **b** Circular map of PCN061 chromosome

We sequenced the genomes of PCN033 and PCN061 using a Roche 454 GS-FLX sequencer. Sequencing of PCN033 and PCN061 generated 30-fold and 33-fold coverage of reads, and produced 113 and 148 large contigs (>500 bp), respectively. The gaps between large contigs were closed by PCR-based sequencing. PCN033 contained a circular chromosome of 4,987,958 bp (Fig. 2a) with an average G + C content of 50.7 %, and plasmids PCN033p1, PCN033p2 and PCN033p3 (Table 2). The PCN033 chromosome was predicted to harbor seven rRNA operons including a duplicate 5S rRNA gene, similar to that in other *E. coli* strains [14], and 85 tRNA genes. We observed 4,838 predicted coding sequences (CDSs) with an average length of 905 bp (Table 2) covering about 89.0 % of the PCN033 chromosome. Among all the protein-coding genes located on the chromosome, approximately 23.8 % (1152/4838) possessed no clear biological function, with 30 genes unique to the PCN033 genome. PCN061 contained a circular chromosome of 4,603,777 bp (Fig. 2b) with an average G + C content of 50.8 %, and plasmids PCN061p1, PCN061p2, PCN061p3, PCN061p4, PCN061p5, and PCN061p6 (Table 2). The PCN061 chromosome was predicted to harbor an extra tRNA gene and share 83 tRNAs compared with the PCN033 chromosome. In total, 4,432 CDSs with an average length of 883 bp covered about 88.0 % of the PCN061 chromosome (Table 2). Among all the protein-coding genes located on the chromosome, approximately 20.3 % (899/4432) genes were hypothetical proteins, with 40 genes unique to the PCN061 genome.

**Table 2:** Overall genome features of PCN033 and PCN061 strains

Characteristic	PCN033	PCN061
Serogroup	O11	O9
Phylogenetic group	D	A
Chromosome size (bp)	4,987,958	4,603,777
GC (%)	50.7	50.8
Chromosome genes (pseudogenes)	4923 (88)	4586 (123)
tRNA number	85	86
rRNA number	22	22
Genomic island (Prophage regions)	29 (6)	12 (2)
Plasmid (bp; gene no.)	PCN033p1 (3,319; 5),	PCN061p1 (2,014; 6),
	PCN033p2 (4,086; 5),	PCN061p2 (5,754; 13),
	PCN033p3 (161,511; 241)	PCN061p3 (6,222; 14),
		PCN061p4 (34,692; 50),
		PCN061p5 (103,644; 142),
		PCN061p6 (145,722; 166)

A phylogenetic tree was generated through sequence concatenation of 325 genes that were conserved in all 56 *E. coli* strains (Fig. 1; Additional file 1: Table S1; Additional file 2: Table S2). PCN033 was assigned to group D and clustered with enteroaggregative *E. coli* 042 and ExPEC UMN026, while PCN061 was assigned to group A and clustered with commensal *E. coli* HS. Among these *E. coli*, pathogenic strains mainly belonged to groups B2 and D, whereas the nonpathogenic strains mainly belonged to groups A and B1 [15, 16]. The phylogenetic positions of *Shigella* strains were mixed with *E. coli* strains (Fig. 1), which is in agreement with the phylogenetic tree produced by Zhang and Lin [17]. Our findings support previous reports which suggested that *Shigella* strains should be included within the genus *Escherichia* [15, 18].

**Fig. 3:**
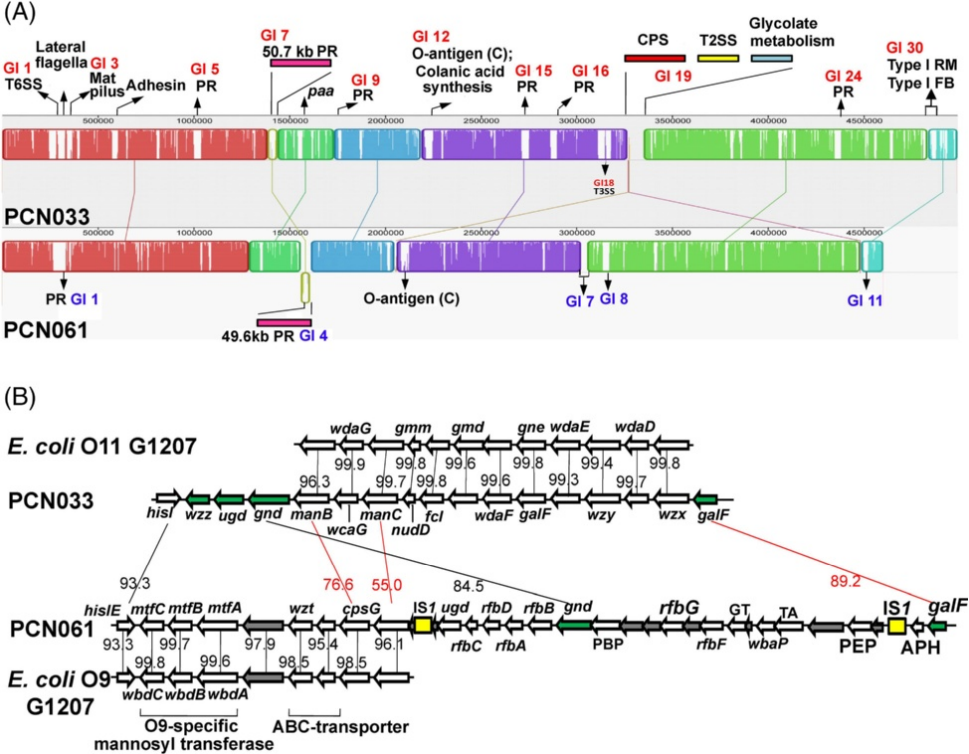
Comparison of genetic organization between PCN033 and PCN061. **a** Complete genomic structure comparison between PCN033 and PCN061. The graph represents an alignment of the colinear blocks, identified by MAUVE, that shared in the two genomes. Each sequence of identically colored blocks represents a collinear set of matching regions. One connecting line links per collinear blocks. Arrows points out the unique regions of PCN033 and PCN061. GI means genomic island. PR means prophage region. RM means restriction-modification system. FB means fimbrial biosythesis. CPS means capsular polysaccharide synthesis. **b** Comparison of gene clusters for O-antigen polysaccharide synthesis between PCN033 and *E. coli* O11 G1207, PCN061 and *E. coli* O9 F719. Arrow represents predicted ORF. Gray arrow represents hypothetical protein gene. Green arrow represents “housekeeping” genes identified as reference. Yellow rectangle represents IS element. Homologous genes in different strains are linked by lines. Black number near by the connecting line means the nucleotide similarity between homologous genes (100 %). Red number means amino acid similarity between homologues (100 %). PBP: polysaccharide biosynthesis protein. GT: glycosyltransferase. TA: tyrosine autokinase. APH: acid phosphatase homologues. PEP: polysaccharide export protein

The PCN033 genome is 384,180 bp longer than that for PCN061. The MAUVE alignment of PCN033 and PCN061 at the nucleotide level identified eight Locally Co-linear Blocks (Fig. 3a). Approximately half of the PCN033 special regions were prophage-containing genomic islands (Fig. 3a). Besides prophage-associated genes, a number of VFs and metabolism-associated genes are located in the special regions of PCN033; these include genes for fimbrial biosynthesis, adhesin, O-antigen polysaccharide synthesis, capsular polysaccharide synthesis, type II secretion system (T2SS), type III secretion system (T3SS), T6SS, the type I R-M system, and the *paa* operon involved in glycolate metabolism. Those regions that likely represent the genetic basis for the virulence differences between PCN033 and PCN061 are further discussed below.

### Comparison of PCN033 with other ExPEC and commensal *E. coli* strains

We chose other nine *E. coli* strains for comparative genomic analysis with PCN033: two commensal *E. coli* strains, K-12 strains MG1655 [GenBank: NC_000913] [19] and W3110 [GenBank: NC_007779] [20]; avian ExPEC strain, APECO1 [GenBank: NC_008563] [8]; six human ExPEC strains, including three UPEC strains, CFT073 [GenBank: NC_004431] [21], UTI89 [GenBank: NC_007946] [22] and UMN026 [GenBank: NC_011751.1] [23], and three NMEC strains, S88 [GenBank: NC_011742] [23], CE10 [GenBank: NC_017646] [24] and IHE3034 [GenBank: NC_017628] [25]. These nine *E. coli* strains are representative of human and avian ExPEC and commensal *E. coli* strains. The main features of the above ten *E. coli* strains are shown in Table 3. Among the ExPEC strains, PCN033 has the smallest chromosome (Table 3). We used ANI value to analyse the pairwise similarities between PCN033 and each of the nine genomes [26] (Table 3). The result showed that PCN033 genome shared most similarities with human UPEC strain UMN026, followed by human MNEC strain CE10 and commensal *E. coli* strains, MG1655 and W3110. Interestingly, PCN033 shared least whole-genome sequence similarities with APEC O1. This supports the notion that some porcine ExPEC strains share more similarity with human ExPEC than avian ExPEC, and suggests a possible relationship between some human and porcine ExPEC. However, additional work is needed to confirm or reject this hypothesis.

**Table 3:** Basic features of selected nine *E. coli* strains and ANI value between these strains with PCN033

Strain	Serotype	Pathotype	Phylogroup	Chromosome				Plasmid size (bp)
				Size (bp)	Total genes	GC (%)	ANI value with PCN033	
PCN033	O11	ExPEC	D	4,987,958	4,923	50.7	-	3,319; 4,086; 161,511
MG1655	OR:H48:K-	Commensal	A	4,641,652	4,497	50.8	97.56	0
W3110	-	Commensal	A	4,646,332	4,436	50.8	97.53	0
CE10	O7:K1	MNEC	F	5,313,531	5,198	50.6	97.63	4,197; 5,163; 1,549; 54,289
S88	O45:K1:H7	MNEC	B2	5,032,268	5,187	50.7	97.2	133,853
IHE3034	O18:K1:H7	MNEC	B2	5,108,383	4,966	50.7	97.13	0
UMN026	O7:K1	UPEC	D	5,202,090	5,089	50.7	98.15	122,301; 33,809
UTI89	-	UPEC	B2	5,065,741	5,127	50.6	97.15	114,230
CFT073	O6:K2:H1	UPEC	B2	5,231,428	5,574	50.5	97.15	0
APECO1	O1:K1:H7	APEC	B2	5,082,025	4,545	50.5	97.12	174,241; 241,387

ANI value of all genome sequence between two strains [26, 99]

**Fig. 4:**
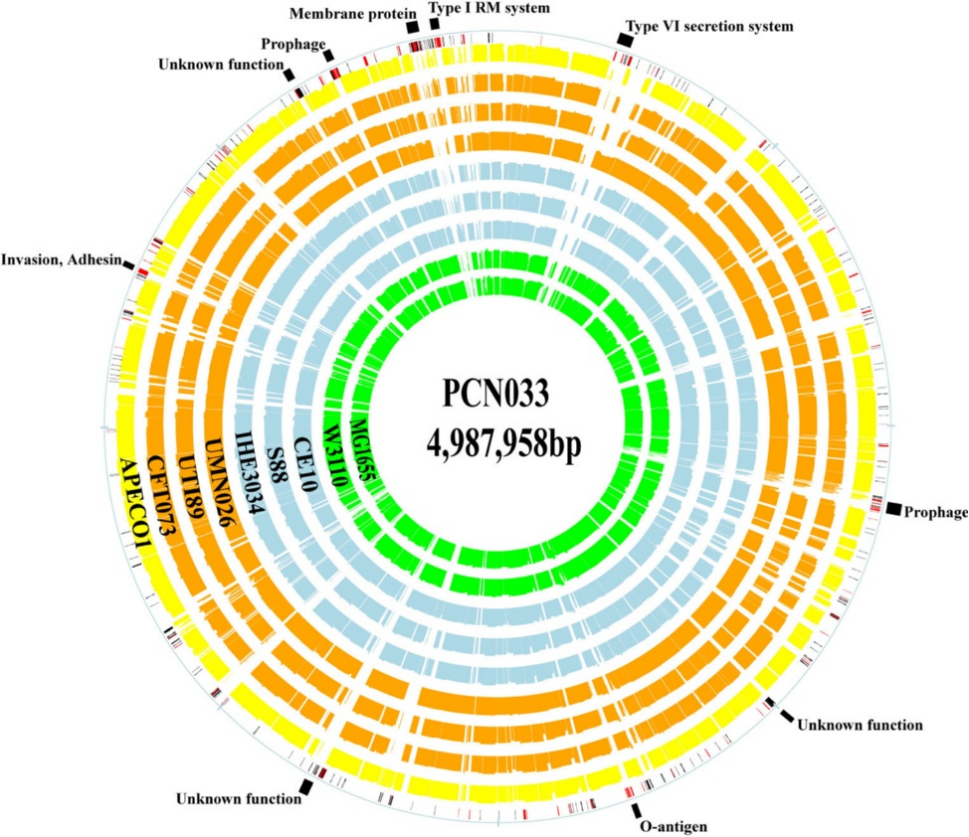
Comparison of PCN033 CDSs with other seven ExPEC strains and two commensal *E. coli* strains. The green circles represent commensal *E. coli* strains, the blue circles represent NMEC strains, the orange circles represent UPEC strains and the yellow is an APEC strain. Special CDSs in PCN033 chromosome compared with other nine *E. coli* strains were showed on the outer second circular, red for CDSs with predicted function, gray for function unknown. The PCN033 special CDSs clustered with similar function were labeled (the outside black boxes)

To identify genomic features specific to PCN033, we initially compared this genome sequence with that of nonpathogenic *E. coli* strains MG1655 and W3110. The comparison identified 24 genomic islands (GIs) (>9 kb) absent in MG1655 and W3110 genomes (Additional file 3: Table S3). The size of the PCN033 special islands varies from 9 kb to 52 kb and 570 kb in all. Many of the islands harbor genes coding for putative virulence factors, phage components and metabolic enzyme, such as type VI secretion system (island I), lateral flagellar (island II), lipopolysaccharide (island X), type III secretion system (island XIII), polysialic acid capsular polysaccharide (island XIV), heme transporter system (island XVI), invasin (island XVII), adhesin (island XXII), prophage (island V-VII, XI, XII, XIX) and metabolic enzyme to break down hydroxyphenylacetic acid (*hpa* operons [27], island XXIV). All-against-all BLASTP comparison of all proteins among PCN033 and other nine *E. coli* strains mentioned above showed that PCN033 had 425 special CDSs (Additional file 4: Table S4, Fig. 4), 68.5 % of which encoded hypothetical proteins. Additionally, PCN033-specific CDSs included genes encoding a type I RM system, type VI secretion system, adhesins, membrane proteins, prophages and O-antigen (Additional file 4: Table S4). Those regions may contribute to this strain’s ability to cause extraintestinal infections and even death in pigs. However, their roles in the pathogenicity of porcine ExPEC will require further study and these special loci in PCN033 should be verified in a larger population of pig ExPEC strains.

### Mobile genetic elements

Most ExPEC VFs that encode fimbriae or adhesins, and confer utilization of alternative nutrients, or resistance to biological stresses are clustered together on GIs known as pathogenicity islands (PAIs) [28, 29]. Using Island Viewer, PCN033 contains a total of 30 GIs (P33GI1–30; Additional file 5: Table S5). Among these, P33GI2, P33GI18 and P33GI19 contain T6SS, T3SS and T2SS, respectively. Additionally, P33GI1, P33GI3, P33GI8, P33GI27 and P33GI30 contain genes for fimbrial biosynthesis, along with P33GI19, P33GI21, P33GI22, and P33GI30, which are GIs containing genes that encode polysaccharide and lipopolysaccharide biosynthesis enzymes, and invasion and type I R-M enzymes. These regions constitute the major PAIs of PCN033. P33GI12 encodes mannose metabolism enzymes, contributing to alternative utilization of nutrients. P33GI14, P33GI23 and P33GI25 include genes associated with antibiotic resistance. In contrast with PCN033, PCN061contains 11 GIs (P61GI1–11; Additional file 5: Table S5). The T2SS is included within P61GI9, while P61GI2 contains tetracycline resistance-encoding genes. The genome of PCN061 contains two prophage regions (P61GI1, P61GI4).

**Fig. 5:**
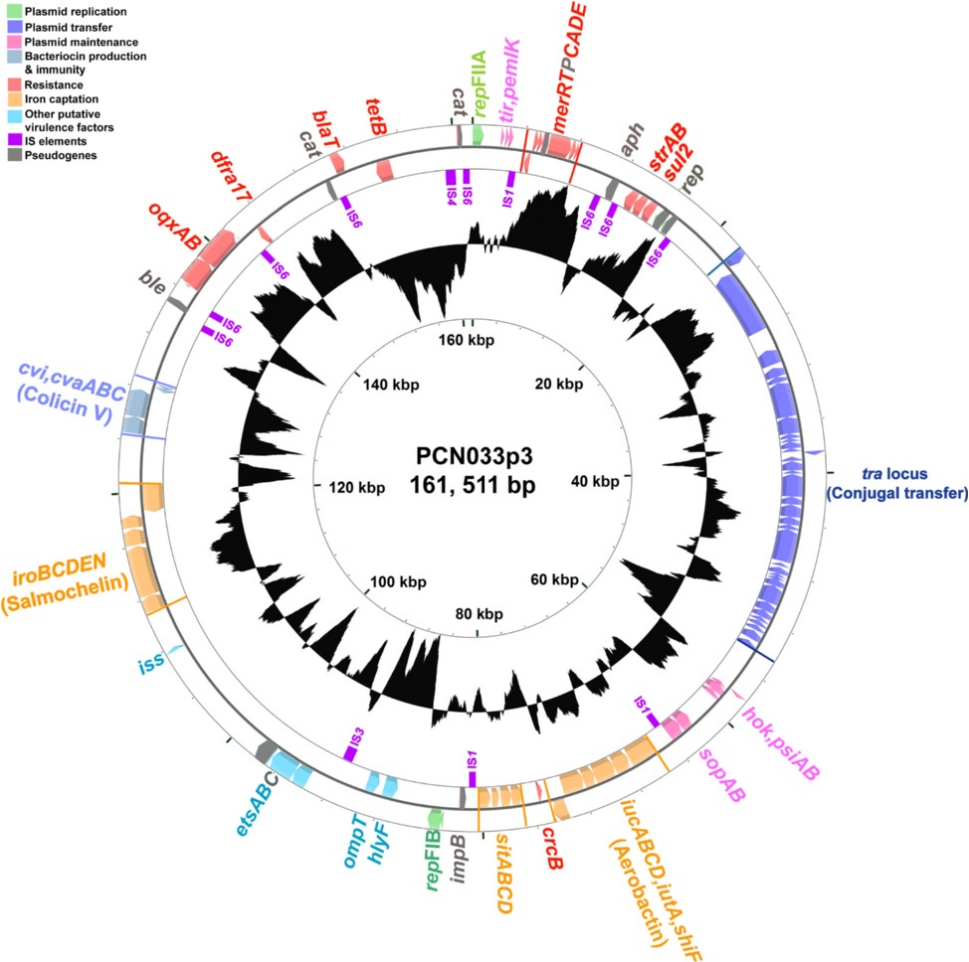
Circular representation of the porcine ExPEC strain PCN033 plasmid PCN033p3. Circles display (from the outside) (1) predicted ORFs transcribed in the clockwise direction, (2) predicted ORFs transcribed in the counterclockwise direction, (3) IS elements (purple), (4) GC skew (G + C/G-C) in a 1,000-bp window, (5) coordinates in kilobase pairs (kbp) from the origin of replication. Genes displayed in circles 1 and 2 are categorized by color as follows: grass green, plasmid replication; dark blue, plasmid transfer; pink, plasmid maintenance; light blue, bacteriocin production and immunity; red, resistance; orange, iron captation; sky blue, other virulence factors; purple, IS elements; gray, pseudogenes

**Fig. 6:**
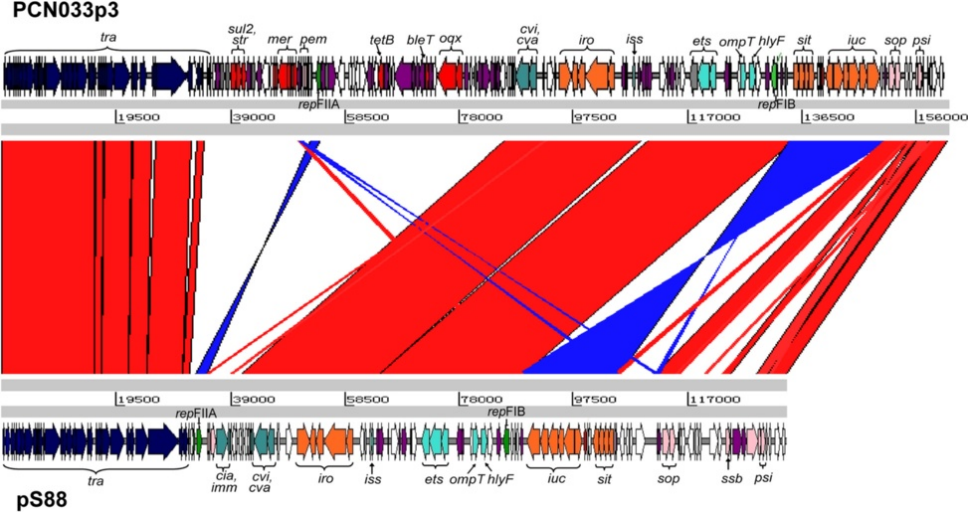
Comparison of plasmid PCN033p3 (161,511 bp) with plasmid pECOS88 (133,853 bp) using the line-plot representation of homologous regions. The *traM* start codon was chosen as the beginning of the two sequences. Genes presented are categorized using the color scheme described in Fig. 5

In addition to the known chromosomally encoded VFs in human ExPECs and APEC [30, 31], many ExPEC VFs are also carried on plasmids [6, 32]. PCN033 harbored three plasmids (Table 2), including the largest (PCN033p3), which contained four replicons. Blast analysis demonstrated that the replicon regions belonged to Incompatibility (Inc) types Q1, FII, FIC and FIB, respectively [33]. Plasmid PCN033p3 carried 241 predicted CDSs, among which 28.2 % (68/241) encoded hypothetical proteins. This plasmid also carried the genes encoding colicin V (ColV) (*cvaA* to *cvaC*, *cvi*), a representative characteristic of ExPEC [34]; a “constant” region of ColV plasmids which includes the RepFIB replicon [35, 36]; three different iron uptake and utilization systems (aerobactin, salmochelin, and the *sitABCD* genes) [37, 38]; an outer membrane protease-encoding gene *ompT* [39]; a novel ABC transport system, *etsABC* [37]; a hemolysin-encoding gene *hlyF* [40]; and the increased serum survival gene *iss* [41]. Additionally, plasmid PCN033p3 also contained numerous resistance genes, including those encoding resistance to mercury (*merR* to *merE*), camphor (*crcB*), trimethoprim (*dfrA17*), kanamycin (*aph*), streptomycin (*strAB*), sulfonamide (*sul2*), bleomycin (*ble*), β-lactams (*bla*_TEM1_), tetracycline (*tetB*), chloramphenicol (*cat*) and olaquindox (*oqxAB*) (Fig. 5) [42]. Many of these resistance-related genes were flanked by insertion sequence (IS) elements; in particular, *cat* (PCN033p3_223) was inserted via an IS*6* element (Fig. 5). This plasmid contained a conjugal transfer region of 41 genes (*traM* to *finO*), that spanned 33,784 bp. The F-like transfer region shares high similarity with that of pECOS88 [GenBank: NC_011747] which is proved to own conjugal transfer ability *via* conjugations experiments (Fig. 6) [43]. Besides the conjugal transfer region, several gene blocks of PCN033p3 sequence are also highly homologous to pECOS88, such as the virulence region including colicin V operon, iron uptake systems (*iro*, *sit* and *iuc* loci), *iss*, *etsABC*, *ompT* and *hlyF* (Fig. 6). As shown in Fig. 6, between the locus *tra* and the virulence region in PCN033p3 lies a region containing mostly resistance-associated genes, such as *sul2*, *strAB*, *mer* operon, *tetB*, *bla*_TEM1_ and *oqxAB*, and genes involved in plasmid stability, *tir*, encoding transfer inhibition protein; *pemIK*, coding for stable plasmid inheritance proteins. This region was absent in pECOS88 (Fig. 6). Plasmid pECOS88, harbored by *E. coli* strain S88 that cause neonatal meningitis, is a key virulence determinant, as it is involved in the ability of S88 to induce high level of bacteremia [43]. The other two PCN033 plasmids only have five unknown functional CDSs respectively. PCN061 possesses six plasmids (Table 2). Blast analysis showed that PCN061p5 belonged to Inc group IncI1, and PCN061p6 was a mosaic plasmid belonging to Inc types IncN, IncFIB and IncX1 [33]. PCN061 also contained several resistance determinants distributed among its plasmids. Plasmid PCN061p3 harbored the resistance genes *sul* and *strAB*. Plasmid PCN061p5 contained the gene *floR*, which encodes resistance to florfenicol/chloramphenicol. Resistance determinants for silver and other heavy metals (*silESRCFBAP*), aminoglycosides (*aph*, *aadA4* and *aac3IIa*), β-lactams (*bla*_TEM1_), and olaquindox (*oqxAB*) were located in PCN061p6. The *sil* gene cluster was similar to that found in pAPEC-O2-R [GenBank : NC_ 006671.1] [44]. Additionally, PCN061p5 encodes the colicin-Ib. Compared with PCN061 plasmids, PCN033 plasmids apparently harbor a greater number of virulence and resistance determinants.

### Other putative virulence factors

#### Adhesins

**Fig. 7:**
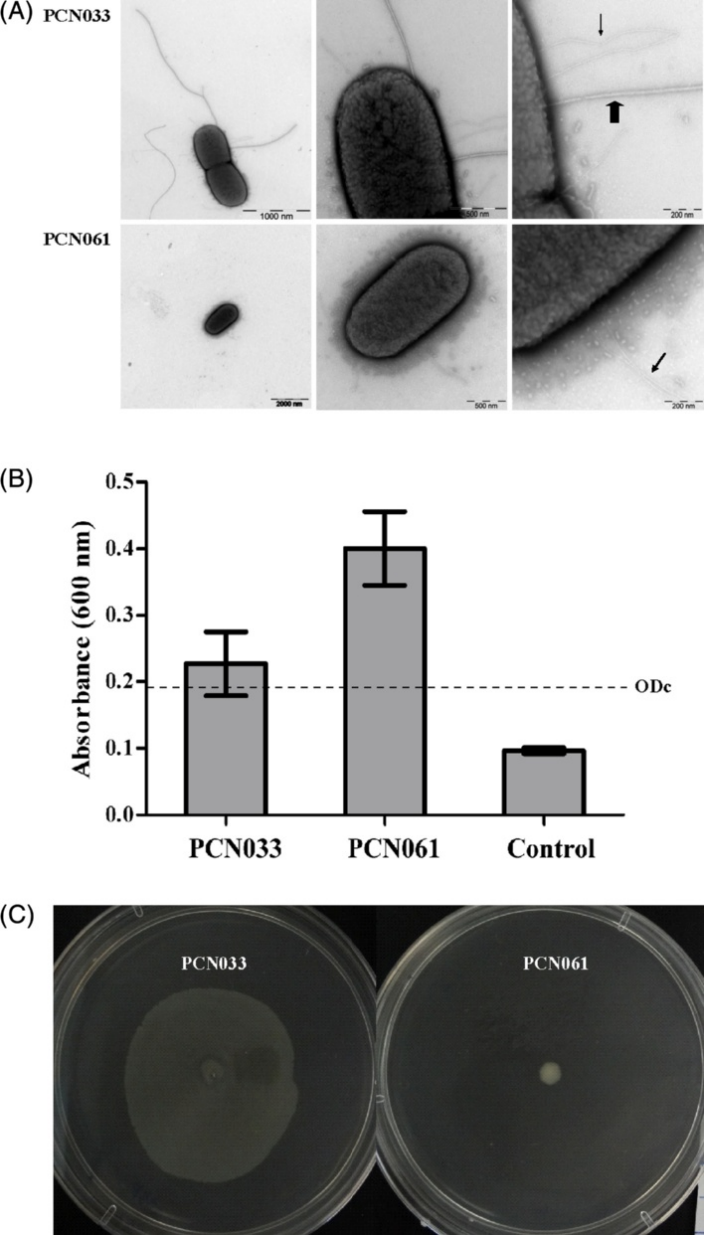
Phenotype analysis of PCN033 and PCN061. **a** Transmission electron micrographs of PCN033 and PCN061 strains. Bacteria were grown overnight at 37 °C in solid medium (TSA). The scale bar indicates the manification size. The scale bars in micrographs of PCN033 were 1000 nm, 500 nm and 200 nm, successively (from left to right). The scale bars in micrographs of PCN061 were 2000 nm, 500 nm and 200 nm, successively (from left to right). Thin arrows indicate fimbriae, thick arrow indicats flagella. **b** Biofilm formation of PCN033 and PCN061 strains on the polystyrene surface. Each strain was tested in three-wells in a 96-well microtiter plate. The optical density of the bacterial biofilm formation were recorded at OD_600nm_ after 24 h incubation. Data are expressed as relative biofilm values (mean ± standard deviation) and representative of three independent experiments. The broken line indicates the cutoff value (ODc = 0.192) for determining a biofilm producer, defined as two times the negative control value. * *P* < 0.05. **c** Swarming motility assay. Assays were performed in triplicate at 37 °C

*E. coli* adhesins are important for determining the bacterium to bind to and colonize the host. Dozens of fimbrial adhesin genes are located in the PCN033 GIs; Yeh-like, Ecp, F9, K99 and type I fimbrial biosynthesis related gene clusters are located on P33GI1, 3, 8, 27 and 30, respectively. In contrast, P61GIs contain no fimbrial adhesin genes, suggesting that the fimbrial adhesins of PCN033 were acquired to adapt towards enhanced colonization and fitness. The pathways involved in assembling fimbriae comprise the chaperone-usher (CU) pathway, the type IV secretion pathway and the extracellular nucleation precipitation pathway [45]. The CU fimbriae found in PCN033 and PCN061 are classified according to Wurpel et al. [46] and Nuccio and Baumler [45]. In the genome of PCN033 and PCN061, gene clusters associated with fimbrial biosynthesis were listed in Table 4. Accordingly, fimbriae were observed in both PCN033 and PCN061 (Fig. 7a). The primary structure of type 3 fimbriae in PCN061p6 shares 93–100 % similarity with that in pMAS2027 [GenBank: NC_013503] and pOLA52 [GenBank: NC_010378]. The expression of type 3 fimbriae in pMAS2027 and pOLA52 determines their biofilm formation [47, 48]. The biofilm formation assay showed that both PCN033 and PCN061 strains were biofilm producers, and that PCN061 exhibited a higher degree of biofilm formation than PCN033 (Fig. 7b).

**Table 4:** Fimbrial gene clusters found in PCN033 and PCN061

Type^α^		Cluster location in PCN033	Cluster location in PCN061
α	Mat(Ecp)	PCN033_0333-0338^a^	-
γ_1_	Smf	PCN033_0593-0597^a^	PCN061_0579-0584^a^
	Ycb	PCN033_1033-1039^a^	PCN061_0970-0976^a^
	F9	PCN033_1642-1647^a^	PCN061_1484-1486
	Type I	PCN033_4812-4820^a^	PCN061_4498-4505^a^
	Yra	-	PCN061_3232-3235^a^
γ_4_	Yeh	PCN033_2280-2283^a^	PCN061_2192-2195^a^
	Yeh-like	PCN033_0019-0021^a^	PCN061_0022
	Yad	PCN033_0142-0148^a^	PCN061_0136
	Mrk(Type III)	-	PCN061p6_067-071^a^
π	Ybg	PCN033_0733-0736^a^	PCN061_0735-0742
	Yfc	PCN033_2517-2522	PCN061_2433-2438^a^
	Yqi	PCN033_3368-3372^a^	PCN061_3092-3094^a^
κ	K99	PCN033_4573	-
Curlin		PCN033_1119-1126^a^	-
Type IV		-	PCN061p5_124-133^a^

Type^α^ based on Wurpel *et al*. [46] and Nuccio and Baumler [45]

“-” indicates the fimbrial gene clusters are absent

^a^Fimbrial gene clusters are intact

### Lipopolysaccharides (LPS) biosynthesis

As an essential structural component of the Gram-negative bacterial outer membrane, LPSs are considered virulence determinants, and help bacteria resist environmental stress. The result of serotyping test presented that PCN033 and PCN061 have an O11 and an O9 type O-antigen, respectively [9]. Previous epidemiological studies of 81 porcine ExPEC isolates in central China showed that O11 is one of the predominant serogroups of porcine ExPECs [11]. PCN033 contains the O-antigen synthesis-related gene cluster, which resembles that for *E. coli* O11 G1207 [GenBank: HQ388393] (Fig. 3b) [49]. In PCN061, part of its O-antigen cluster is identical with that in the O9 *rbf* gene cluster of *E. coli* F719 [GenBank: D43637] (Fig. 3b) [50, 51]. The genetic analyses match the result of serotyping test.

### Capsular polysaccharide (CPS)

Surface-associated CPSs are involved in protecting microorganisms from desiccation, promoting adhesion, and resisting the host immune system [52, 53]. Capsules of *E. coli* were separated into four different groups [54]. Sequence alignment showed PCN033 capsule belongs to group 2. However, the PCN033 capsular region also encodes four hypothetical proteins and a hydrolase that show no similarity with products in other known group 2 capsule regions. *E. coli* K1 which also belong to group 2 capsule had the ability to invade brain microvascular endothelial cells [55]. In PCN061, a single gene (PCN061_1020) involved in CPS biosynthesis was found; this gene encodes a protein with a capsule biosynthesis GfcC domain (Pfam: PF06251) which plays a role in group 4 capsule biosynthesis [56].

### Iron acquisition and utilization

**Fig. 8:**
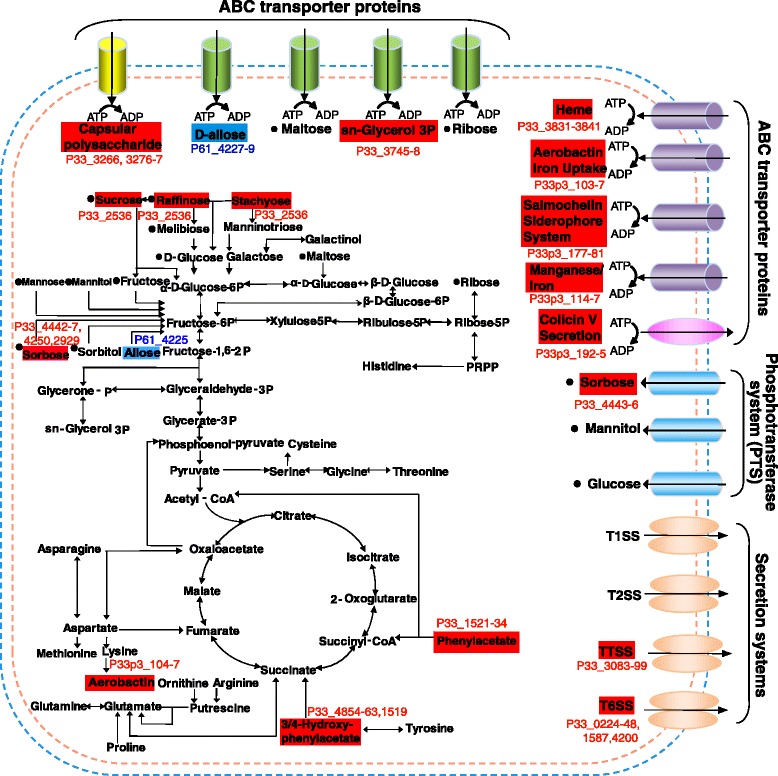
Differences of predicted metabolism and transport between PCN033 and PCN061. Solid square frames point out the differences of metabolism and transport between PCN061 and PCN033. Red and blue solid square frame points out the substance metabolised or system present only in PCN033 and PCN061, respectively (by identifing whether the genes associated with the substance metabolism or system are present in the genome, black dots note the metabolism proved by biochemical experiments). Arrows indicate the direction of transport. P33 means PCN033; P61 means PCN061; P33p3 means PCN033p3

Iron transport systems are known to play an important part in bacterial pathogenesis [57, 58]. For PCN033 and PCN061, gene clusters involved in hydroxamate, enterobactin and ferric enterobactin-mediated iron uptake systems have been revealed (Fig. 8). Additionally, genes for ferrochelatase (HemH), ferritin proteins, ferric uptake regulator (Fur), ferric reductase, ferrous iron transporter (EfeUOB) and TonB systems also exist in the two strains, suggesting they have the capacity to maintain iron homeostasis allowing them to inhabit a host. Compared with PCN061, PCN033 harbors four extra iron uptake systems including a chromosome-encoded heme transporter system (Chu) and three plasmid-associated siderophore iron uptake and ABC iron transport systems (aerobactin, salmochelin, and the *sitABCD* genes).

### Secretion Systems

Secretion systems are necessary for the transport of proteins across the cell envelope, mediating interactions between bacteria, their hosts, and the surrounding microenvironment [59]. Differences between PCN033 and PCN061 secretion systems are mainly focused upon the presence of T3SSs and T6SSs (Fig. 8).

T3SSs are essential components of two complicate bacterial apparatuses: the flagellum, the motility equipment, and the non-flagellar T3SSs (NF-T3SSs) which deliver effectors into the cytosol of host cells [60]. According to previous reports, two flagella systems exist in *E. coli* [61]. The Flag-1 flagellum cluster is associated with peritrichous (lateral) flagellum biosynthesis and identified in PCN033 and PCN061. In PCN061, *flhA* and *flhD* which encode a peritrichous flagellum biosynthesis protein and transcriptional activator of Flag-1 operons respectively, both contain frame-shift mutations [62]. The frame-shift mutation in *flhD* of *Yersinia pestis* is associated with its loss of motility [63], suggesting that the Flag-1 system is not functional in PCN061. In contrast, genes involved in the Flag-1 system are all intact in PCN033. In addition to the conventional Flag-1 cluster, PCN033 contains a Flag-2 gene cluster, which has been found in around 20 % of *E. coli* strains [64]. This gene cluster was first reported in *E. coli* O42; however, the *lfgC* gene that encodes a FlgC-like rod protein in the Flag-2 system contains a frame-shift mutation. Ren et al. suspected that a frame-shift mutation in *lfgC* probably inactivated the Flag-2 system in *E. coli* O42 [64]. The *lfgC* gene in PCN033 does not contain any mutations. Additionally, PCN033 LafK protein in Flag-2 contains a full-length Sigma54 activating domain [Pfam: PF00158]. Furthermore, consensus σ^54^ sites (TGGCAC-N_5_-TTGC) are identified upstream of both *lfgB* and *lafB* translation start codons. These motifs suggest that the Flag-2 system is functional in PCN033, while only four genes (PCN061_0225–0228) involved in the Flag-2 system are found in PCN061. The results of transmission electron microscopy and swarming motility assays (Fig. 7a, c) showed that PCN033 was indeed able to produce flagella and motile, while PCN061 did not possess flagella. Two NF-T3SSs were found in *E. coli* strains: *E. coli* type III secretion system 1 (ETT1); and *E. coli* type III secretion system 2 (ETT2) [65]. ETT1 was absent in both PCN033 and PCN061, while an ETT2 gene cluster (PCN033_3083–3099) was found in PCN033 and located in P33GI18. This ETT2 gene cluster is highly homologous with that in meningitis-causing *E. coli* K1 strain EC10 (O7:K1), but contained less deletions and no insertions. EC10 mutants lacking ETT2 and/or *eivA* exhibited significant defects in invasion and intracellular survival in human brain microvascular endothelial cells (HBMECs) compared with parental strains [66], suggesting that ETT2 in PCN033 contributes to its pathogenicity. ETT2 is absent from the PCN061 genome.

T6SSs are the most recently identified protein secretion systems of Gram-negative bacteria [67, 68]. Although the gene order is diverse among different organisms, products of conserved genes compose the core elements of T6SS, including effector proteins, hemolysin coregulated protein (Hcp), valine-glycine repeat protein G (VgrG), intracellular multiplication factor (IcmF) and the chaperone ClpB [68, 69]. These core components are all found in PCN033, with IcmF (PCN033_0227), ClpB (PCN033_0230) and VgrG (*vgrG1*, PCN033_0247) sharing 98.0, 97.8 and 95.8 % identity respectively with the corresponding proteins in *E. coli* Sakai. PCN033 Hcp1 (*hcp1*, PCN033_0225) is 100 % identical to Hcp-like proteins in *Shigella sonnei* Ss046 and *E. coli* EDL933 strains [68]. PCN033 Hcp2 (*hcp2*, PCN033_0245) shares 94.2 % identity with the Hcp2 (EvfV) protein of the meningitis-causing *E. coli* K1 strain RS218. Previous research has shown that Hcp2 of *E. coli* RS218 contributes to its interaction with HBMECs [70]. Outside of this T6SS gene cluster, we identified a third Hcp (*hcp3*, PCN033_4200) that shared 32.2 % identity with Hcp1 and another VgrG (*vgrG2*, PCN033_1587); this Hcp protein exhibited 99 % identity with VgrG1. In contrast, we were unable to identify T6SS-associated genes in PCN061.

### Predicted metabolic pathways

Complete sets of genes encoding enzymes necessary for glycolysis and gluconeogenesis, the tricarboxylic acid cycle, and the pentose phosphate and Entner-Doudoroff pathway, were identified in the genomes of PCN033 and PCN061 (Fig. 8). Bacterial uptake of sugars mainly occurs through specific phosphotransferase systems (PTSs) and ATP-binding cassette (ABC) transport systems. In the genomes of PCN061 and PCN033, predicted sets of genes encoding PTSs and ABC transport systems were identified [71–74] (Fig. 8). The CAP (*crp*, PCN033_3662; PCN061_3439)-cAMP (*cyaA*, PCN033_4172; PCN061_3937) system regulates the transcription of multiple sugar utilization operons and was found in PCN033 and PCN061 [75]. The Cra (*fruR*, PCN033_0087; PCN061_0079) transcription factor plays a key role in balancing enzyme levels for carbon metabolism [76], and was also seen in strains PCN033 and PCN061, suggesting their ability to metabolize those sugars as carbon source. These metabolic characteristics of the two strains were verified by an API 20E test (Additional file 6: Table S6) and other biochemical tests (Fig. 8).

Sucrose is the most abundant disaccharide in most environments. The ability to utilize sucrose as a sole carbon source is a highly variable phenotype among enteric bacteria [77]. This characteristic is dependent upon the presence of the csc regulon comprising three genes for a sucrose symporter (CscB, PCN033_2534), a sucrose hydrolase (CscA, PCN033_2536) and fructokinase (CscK, PCN033_2535), and was first reported in *E. coli* EC3132. In EC3132, sucrose is transported into cells by a sucrose:H+ symporter encoded by *cscB*. Jahreis et al. reported that *csc* genes optimally adapt to new hosts [77]. This csc regulon exists in PCN033 but not in PCN061, and the resulting metabolic difference in sucrose utilization was proved through API 20E tests (Additional file 6: Table S6). D-allose is an analog of D-ribose, a key component of DNA and RNA, that can be converted into fructose-6-phosphate [78]. PCN033 is possibly unable to catabolize allose because of the absence of the *alsBACEK* regulon [79]. The *rbsDACBKR* regulon (PCN033_4126–4131) contributes to the metabolism of ribose, and was identified in PCN033. Both regulons were seen in PCN061. The *glc* gene locus is associated with glycolate utilization in *E. coli*, and is known to contain *glcB* (PCN033_3294), which encodes malate synthase G; *glcC* (PCN033_3299), which encodes the *glc* regulator protein; *glcA* (PCN033_3293) encoding a glycolate transporter; and *glcDEFG* (PCN033_3298–3295), which are required for glycolate oxidase activity [80]. The presence of the *glc* gene locus in PCN033 suggests that this strain can use glycolate as another source of carbon; PCN061 did not contain this gene locus.

Vieira et al. conducted an analysis of core- and pan-metabolism in 29 *E. coli* strains, and observed that most commensal strains were able to degrade phenylacetate and phenylethylamine courtesy of the *paa* transcription unit. This particular transcription unit was absent from IPEC and most ExPECs [27, 81]. However, *paa* (PCN033_1521–1534) was found in PCN033, which is inconsistent with previous observations. Besides the ability to degrade phenylacetate and phenylethylamine, PCN033 may also possess the ability to break down 3- and 4-hydroxyphenylacetic acids as it contains the *hpaCBAXIHFDBGR* (PCN033_4854–4863, 4865) gene cluster [27]. PCN061 does not harbor these two gene clusters that are involved in aromatic compound degradation.

## Conclusions

The first full genomic analysis of porcine ExPEC strain demonstrates that porcine ExPEC strain PCN033 shared many similarities with human ExPEC strains. Previous studies have shown that some APEC strains share similarities with human ExPEC strains [8]. These indicated that a possible foodborne link between animal and human ExPEC strains exists, and that animal ExPEC isolates may be a reservoir for human ExPEC [7, 8]. Comparative analysis of the virulent porcine ExPEC strain PCN033 and non-pathogenic porcine *E. coli* strain PCN061 showed that most genomic differences were in the mobile genetic elements of PCN033. Several virulence-associated phenotypic differences in PCN033 and PCN061 strains were verified, and most of the phenotypic differences observed corresponded with genotypic differences. Compared with PCN061, PCN033 has flagella and significantly stronger swarming motility. *E. coli* motility and the presence of flagella impact biofilm architecture [82]; however, the gene loci affecting biofilm formation in PCN033 and PCN061 were diverse and numerous, and we could not easily infer biofilm formation ability of the two strains purely by analyzing genomic sequences. According to our experimental results, PCN061 exhibited a higher degree of biofilm formation than PCN033. During the pathogenesis, bacteria require a balance between adherence, colonization and pervasion. The strong motility of PCN033 results in a reduced ability to form biofilms. Strain PCN033 contained more carbon source utilization genes, and was able to metabolize more carbon substrates. Comparison of PCN033 genome with other nine characteristic *E. coli* genomes revealed 425 PCN033-special CDSs. Genes of interest in this subset including those encoding type I R-M system, T6SS and membrane-associated proteins. Analysis of the T6SS in PCN033 revealed that the core elements of T6SS were intact. These special characteristics of PCN033 are worth further studying. Our genetic and phenotypic analyses of PCN033 have improved our understanding of the pathogenic mechanisms of porcine ExPEC strains and will hopefully lead to the development of new strategies for the prevention and treatment of porcine ExPEC infections.

## Methods

### Ethical statement

The infection experiment was performed based on the International Council for Laboratory Animal Science Ethical Guideline for Researchers (1956). The animal experiment was conducted in Wuhan keqian Animal Biological Products Co., Ltd which obtained the experimental animal use license set by Science and Technology Department of Hubei province. The use license No. of our animal experiment is 00132619.

### Strains and culture

*E. coli* strain PCN033 was isolated from the brain of a diseased pig from Hunan Province, China [10]. *E. coli* strain PCN061 was isolated from the lung of a diseased pig from Hunan Province, China. These two strains were routinely cultured in Luria–Bertani (LB) medium at 37 °C. According to Johnson et al. [12] and Ding et al. [11], ExPECs were defined as *E. coli* isolates containing two or more virulence markers: *papA*/*papC*, *sfa*/*foc*, *afa*/*dra*, *kpsMTII* and *iutA*. PCN033 and PCN061 were examined in PCR for the presence of the above virulence markers. Serotyping, phylogenetic grouping and virulence analysis in mice model of these two strains were performed in previous study [9]. The serotypes of these strains were identified by serum agglutination assay using specific O-antigen antiserum in the China Institute of Veterinary Drugs Control, Beijing, China. The phylogenetic groups of PCN033 and PCN061 strains were determined based on PCR detection of the *chuA* and *yjaA* genes and DNA fragment TSPE4.C2 [83].

### Pathogenicity test in pig

We used 15 high-health-status pigs (4–5 weeks of age) to investigate the virulence of ExPEC strain PCN033. Twelve piglets were evenly assigned to PCN033 and PCN061 group (six pigs per group) and the remaining three were allocated to the PBS negative control group. Prior to the infection, the serum of pigs were tested negative by ELISA for PCN033 strain. Piglets in the PCN033 and PCN061 groups were inoculated with 8 × 10^8^ CFU (1 ml) of each strain by ear vein infection. Three piglets infected with 1 ml PBS were served as negative control. All piglets were observed for morbidity and mortality for a week after infection. The remaining surviving piglets were euthanized. Meanwhile, bacteria were recovered from blood samples collected from the jugular vein before piglets were moribund or euthanized, and used to identified as *E. coli* by plating onto MacConkey selection Agar and 16S rRNA amplification. The infection experiment was performed based on the International Guiding Principles for Biomedical Research Involving Animals (1985).

### DNA extraction and sequencing

Total genomic DNA was isolated from 15 ml of overnight culture using a DNEasy Blood & Cell Culture DNA Mini Kit (Qiagen, Hilden, Germany). The genomes of PCN033 and PCN061 were sequenced using a Roche 454 (GS FLX Tianium) system (http://www.454.com/). Sequence reads were assembled with the Newbler *de novo* assembler package. The relationship between contigs was displayed using ContigScape [84], determined by sequence alignment analysis and verified by PCR. Gaps between neighboring contigs were closed by sequencing PCR products using an ABI 3730 DNA sequencer (Applied Biosystems, Foster City, CA). The Phred/Phrap/Consed software was used for primer design, genome assembly, edition and quality assessment (http://www.phrap.org/consed/consed.html), and low quality regions of the genome were resequenced.

### Gene prediction, annotation, and comparative analysis

Putative open reading frames (ORFs) were predicted using Glimmer [85] and GeneMark [86]. A protein database was constructed based on the 48 *E. coli* genomes available in GenBank (Additional file 1: Table S1). The predicted proteins were searched for against the protein database using BLASTP [87]. Predicted CDSs that did not have any matches, or only weak matches (E-value ≥ 1*E*^−10^, or amino acid sequence identity <40 %, or coverage of the protein <60 %) in the local protein database were compared with GenBank’s non-redundant protein database (http://www.ncbi.nlm.nih.gov/genbank/). Predicted CDSs were also searched against COG (Clusters of Orthologous Groups of proteins; http://www.ncbi.nlm.nih.gov/COG/ [88]) and KEGG (Kyoto Encyclopedia of Genes and Genomes; http://www.genome.jp/kegg/ [89]) for functional annotation and further function assignment. All predicted CDSs were compared with those in *E. coli* strains K-12 MG1655 [GenBank : NC_000913], HS [GenBank: NC_009800] and APECO1 [GenBank: NC_008563] using the tBLASTn algorithm to determine pseudogenes. Transfer RNA (tRNA) and ribosomal RNA (rRNA) genes were predicted using tRNAscan-SE [90] and RNAmmer (http://www.cbs.dtu.dk/services/RNAmmer/ [91]), respectively. Predicted ORFs that overlap with rRNA or tRNA genes were removed. ISs were annotated using BLASTN against the IS finder database [92]. GIs were predicted by Island Viewer [93], and genome circular maps were generated using GenomeViz [94]. Comparative genomic analyses of PCN033 and PCN061 was conducted using Mauve 2.3.1 genome alignment software [95]. ANI value was used to analyse the similarities between two whole-genome sequences [26]. Genomic comparison of porcine ExPEC strain PCN033 with other 7 available ExPEC and 2 commensal *E. coli* strains were based on protein-coding sequences. All-against-All BLASTP for amino acids was used to assign orthologs (E-value ≤1E^−10^, identity ≥60 %, coverage ≥80 %).

To generate the phylogenetic tree of *Escherichieae* strains, we identified orthologous CDSs that were conserved in all 56 fully sequenced strains of *E. coli*, *Shigella*, and *Escherichia fergusonii*, using BLASTClust (http://www.ncbi.nlm.nih.gov/Web/Newsltr/Spring04/blastlab.html) (Additional file 1: Table S1; Additional file 2: Table S2) (>90 % identity with the same length). DNA sets of 325 CDSs from each of the 56 strains were concatenated and used to generate a phylogenetic tree by the neighbor-joining method with 100 bootstrap iterations, using MEGA5 software [96].

### Motility assays

Swarming motility assays were conducted as previously described [97]. The swarming motility plates were prepared with 0.5 % agar, 1.0 % tryptone, 0.5 % Yeast Extract, 0.5 % NaCl and 0.5 % D-(+)-glucose. The plates were photographed after 16-h incubation at 37 °C.

### Biofilm formation assays

Bacterial biofilm formation was assessed using crystal violet as previously described, with some modifications [98]. The wells of a sterile 96-well microplate were filled with 200 μl of LB medium; 2 μl of overnight culture was added to each well. Each strain was tested in triplicate. Wells containing sterile LB medium were treated as negative controls. The plate was covered and incubated at 37 °C for 24 h without shaking. Planktonic cells in wells were aspirated, and washed twice with 200 μl of sterile PBS. Attached cells were stained with 200 μl of 1 % (w/v) crystal violet (Biosharp, Hefei, China) for 15 min at room temperature. Wells were rinsed twice with 200 μl of sterile PBS, and plates dried at 37 °C for 30 min. Stained adherent biofilms were solubilized with 200 μl of 33 % (v/v) glacial acetic acid. To quantify, the optical density (OD) at 600 nm (OD_600_) of each well was determined using a Synergy™ HT Multi-Detection Reader (BioTed Instruments, Winooski, VT). All tests were independently performed three times and the results averaged. The cutoff value for determining a biofilm producer was defined as 2-fold greater than the negative control [97].

### Statistical analysis

Distribution differences in the functional categories of CDSs assigned according to COG between PCN033 and PCN061 strains were analyzed using Pearson’s χ^2^ tests. Differences of biofilm formation ability were determined using Mann–Whitney-Wilcoxon tests. A *P*-value of 0.05 was considered statistically significant.

### Accession numbers

The annotated complete genome sequences were deposited in GenBank database with accession numbers CP006632 (*E. coli* PCN033), CP006633 (plasmid PCN033p1 of *E. coli* PCN033), CP006634 (plasmid PCN033p2 of *E. coli* PCN033), CP006635 (plasmid PCN033p3 of *E. coli* PCN033), CP006636 (*E. coli* PCN061), CP006637 (plasmid PCN061p1 of *E. coli* PCN061), CP006638 (plasmid PCN061p2 of *E. coli* PCN061), CP006639 (plasmid PCN061p3 of *E. coli* PCN061), CP006640 (plasmid PCN061p4 of *E. coli* PCN061), CP006641 (plasmid PCN061p5 of *E. coli* PCN061) and CP006642 (plasmid PCN061p5 of *E. coli* PCN061), respectively.

## Additional files

Additional file 1: Table S1. Genomes used for phylogenetic and *Escherichia coli* protein database construction (DOC 86 kb) Additional file 2: Table S2. Core genome content used to construct phylogenetic tree (XLSX 225 kb) Additional file 3: Table S3. Islands in PCN033 but absent from MG1655 or W3110 (XLS 39 kb) Additional file 4: Table S4. 425 special CDSs in PCN033 (XLSX 29 kb) Additional file 5: Table S5. Overview of the genomic islands in PCN033 and PCN061 (XLSX 14 kb) Additional file 6: Table S6. Biochemical characteristics of PCN033 and PCN061 strains with API 20E test strips (DOC 40 kb)

## Abbreviations

ExPEC: Extraintestinal pathogenic *Escherichia coli*
R-M: Restriction-modification
T6SS: Type VI secretion system
IPEC: Intestinal pathogenic *E. coli*
UPEC: Uropathogenic *E. coli*
NMEC: Newborn meningitis-causing *E. coli*
APEC: Avian pathogenic *E. coli*
CDSs: Coding sequences
T2SS: Type II secretion system
T3SS: Type III secretion system
GIs: Genomic islands
Inc: Incompatibility
ColV: Colicin V
CU: Chaperone-usher
LPS: Lipopolysaccharides
CPS: Capsular polysaccharide
NF-T3SSs: Non-flagellar T3SSs
ETT1: *E. coli* type III secretion system 1
ETT2: *E. coli* type III secretion system 2
HBMECs: Human brain microvascular endothelial cells
PTSs: Phosphotransferase systems
ABC: ATP-binding cassette
LB: Luria–Bertani
ORFs: Open reading frames
