# Supplementary material for: Genome analysis and in vivo virulence of porcine extraintestinal pathogenic Escherichia coli strain PCN033
Source: BMC Genomics. 2015 Sep 21;16(1):717. doi: 10.1186/s12864-015-1890-9 (PMC4578781; doi:10.1186/s12864-015-1890-9)
Supplement: Additional file 1: Table S1. — Genomes used for phylogenetic and Escherichia coli protein database construction (DOC 86 kb) [file 12864_2015_1890_MOESM1_ESM.doc]

**Table S1. Genomes used for phylogenetic and *Escherichia coli* protein database construction**

| **Strains** | **Abbreviation** | | **Origin***#* | | **GenBank Accession No.** | |
| --- | --- | --- | --- | --- | --- | --- |
| *Escherichia coli* str.‘clone D i14’*** | i14 | | Human | | NC_017652 | |
| *Escherichia coli* str.‘clone D i2’* | i2 | | Human | | NC_017651 | |
| *Escherichia coli* CFT073* | CFT073 | | Human | | NC_004431 | |
| *Escherichia coli* ABU83972* | ABU83972 | | Human | | NC_017631 | |
| *Escherichia coli* ED1a* | ED1a | |  | | NC_011745 | |
| *Escherichia coli* O83:H1 str.NRG 857C* | NRG857C | |  | | NC_017634 | |
| *Escherichia coli* LF82* | LF82 | |  | | NC_011993 | |
| *Escherichia coli* S88* | ECS88 | | Human | | NC_011742 | |
| *Escherichia coli* APEC O1* | APECO1 | | Avian | | NC_008563 | |
| *Escherichia coli* IHE3034* | IHE3034 | | Human | | NC_017628 | |
| *Escherichia coli* UM146* | UM146 | |  | | NC_017632 | |
| *Escherichia coli* UTI89* | UTI89 | | Human | | NC_007946 | |
| *Escherichia coli* 536* | 536 | | Human | | NC_008253 | |
| *Escherichia coli* O127:H6 str.E2348/69* | E2348/69 | |  | | NC_011601 | |
| *Escherichia coli* NA114* | ECNA114 | | Human | | NC_017644 | |
| *Escherichia coli* SE15* | SE15 | |  | | NC_013654 | |
| *Escherichia coli* SMS-3-5* | SMS35 | |  | | NC_010498 | |
| *Escherichia coli* O7:K1 str.CE10* | CE10 | | Human | | NC_017646 | |
| *Escherichia coli* IAI39* | IAI39 | | Human | | NC_011750 | |
| *Escherichia coli* 042* | EC042 | |  | | NC_017626 | |
| *Escherichia coli* UMN026* | UMN026 | | Human | | NC_011751 | |
| *Escherichia coli* O55:H7 str.RM12579* | RM12579 | |  | | NC_017656 | |
| *Escherichia coli* O55:H7 str.CB9615* | CB9615 | |  | | NC_013941 | |
| *Escherichia coli* O157:H7 str.EC4115* | EC4115 | |  | | NC_011353 | |
| *Escherichia coli* O157:H7 str.TW14359* | TW14359 | |  | | NC_013008 | |
| *Escherichia coli* O157:H7 str.EDL933* | EDL933 | |  | | NC_002655 | |
| *Escherichia coli* Xuzhou21* | Xuzhou21 | |  | | NC_017906 | |
| *Escherichia coli* O157:H7 str.Sakai* | Sakai | |  | | NC_002695 | |
| *Escherichia coli* APEC O78* | APECO78 | | Avian | | NC_020163 | |
| *Escherichia coli* O111:H- str.11128* | 11128 | |  | | NC_013364 | |
| *Escherichia coli* O26:H11 str.11368* | 11368 | |  | | NC_013361 | |
| *Escherichia coli* E24377A* | E24377A | |  | | NC_009801 | |
| *Escherichia coli* 55989* | 55989 | |  | | NC_011748 | |
| *Escherichia coli* O103:H2 str.12009* | 12009 | |  | | NC_013353 | |
| *Escherichia coli* IAI1* | IAI1 | |  | | NC_011741 | |
| *Escherichia coli* SE11* | SE11 | |  | | NC_011415 | |
| *Escherichia coli* KO11FL* | KO11FL | |  | | NC_017660 | |
| *Escherichia coli* W* | W | |  | | NC_017664 | |
| *Escherichia coli* HS* | EcHS | |  | | NC_009800 | |
| *Escherichia coli* B str.REL606* | REL606 | |  | | NC_012967 | |
| *Escherichia coli* BL21(DE3)* | BL21 | |  | | NC_012971 | |
| *Escherichia coli* UMNK88* | UMNK88 | |  | | NC_017641 | |
| *Escherichia coli* ETEC H10407* | H10407 | |  | | NC_020163 | |
| *Escherichia coli* BW2952* | BW2952 | |  | | NC_012759 | |
| *Escherichia coli* str.K-12 substr.DH10B* | ECDH10B | |  | | NC_010473 | |
| *Escherichia coli* DH1* | EcDH1 | |  | | CP001637 | |
| *Escherichia coli* str.K-12 substr.W3110* | W3110 | |  | | NC_007779 | |
| *Escherichia coli* str.K-12 substr.MG1655* | MG1655 | |  | | NC_000913 | |
| *Escherichia coli* PCN033 | PCN033 | |  | | CP006632 | |
| *Escherichia coli* PCN061 | PCN061 | |  | | CP006636 | |
| *Shigella boydii* Sb227 | Sb227 | |  | | NC_007613 | |
| *Shigella dysenteriae* Sd197 | Sd197 | |  | | NC_007606 | |
| *Shigella flexneri* 2a str. 301 | Sf301 | |  | | NC_004337 | |
| *Shigella flexneri* 5 str. 8401 | Sf8401 | |  | | NC_008258 | |
| *Shigella sonnei* Ss046 | Ss046 | |  | | NC_007384 | |
| *Escherichia fergusonii* ATCC 35469 | EFER | |  | | NC_011740 | |
| * notes the *E. coli* genome used for *E. coli* protein database construction  # As the main organism of this study was ExPEC, we only listed the origin of ExPEC | | | | | |  |
| Strains with underlines are previously reported ExPECs | |  | |  | |  |
