# Supplementary material for: Genome analysis and in vivo virulence of porcine extraintestinal pathogenic Escherichia coli strain PCN033
Source: BMC Genomics. 2015 Sep 21;16(1):717. doi: 10.1186/s12864-015-1890-9 (PMC4578781; doi:10.1186/s12864-015-1890-9)
Supplement: Additional file 6: Table S6. — Biochemical characteristics of PCN033 and PCN061 strains with API 20E test strips (DOC 40 kb) [file 12864_2015_1890_MOESM6_ESM.doc]

**Table S6 Biochemical characteristics of PCN033 and PCN061 strains with API 20E test strips**

|  | PCN033 | PCN061 |
| --- | --- | --- |
| ONPG | + | + |
| ADH | - | - |
| LDC | + | + |
| ODC | - | - |
| CIT | - | - |
| H2S | - | - |
| URE | - | - |
| TDA | - | - |
| IND | - | - |
| VP | - | - |
| GEL | - | - |
| GLU | + | + |
| MAN | + | + |
| INO | - | - |
| SOR | + | + |
| RHA | + | + |
| SAC | + | - |
| MEL | + | + |
| AMY | - | - |
| ARA | + | + |

API 20E test of PCN033 and PCN061 strains. All the data were collected after 20 h incubation at 37°C. ONPG, β-galactosidase; ADH, arginine dihydrolase; LDC, lysine decarboxylase; ODC, ornithine decarboxilase; CIT, citrate utilization; H2S, H_2_S production; URE, urease; TDA, tryptophane deaminase; IND, indole production; VP, Voges–Proskauer; GEL, gelatinase; GLU, glucose; MAN, mannitol; INO: inositol; SOR: sobitol; RHA, rhamnose; SAC; saccharose; MEL, melibiose; AMY: amygdalin; ARA, arabinose.
